# Role of maraviroc and/or rapamycin in the liver of IL10 KO mice with frailty syndrome

**DOI:** 10.1371/journal.pone.0286201

**Published:** 2024-01-10

**Authors:** Laura Pérez-Martínez, Lourdes Romero, Eva M. Verdugo-Sivianes, Sandra Muñoz-Galván, Susana Rubio-Mediavilla, Ana Amiama-Roig, Amancio Carnero, José-Ramón Blanco

**Affiliations:** 1 Centro de Investigación Biomédica de La Rioja (CIBIR), Logroño, Spain; 2 Instituto de Biomedicina de Sevilla, IBIS, Hospital Universitario Virgen del Rocío, Consejo Superior de Investigaciones Científicas, Universidad de Sevilla, Sevilla, Spain; 3 CIBERONC, Instituto de Salud Carlos III, Madrid, Spain; 4 Servicio de Anatomía Patológica, Hospital San Pedro, Logroño, Spain; 5 Servicio de Enfermedades Infecciosas, Hospital Universitario San Pedro, Logroño, Spain; Indiana University School of Medicine, UNITED STATES

## Abstract

Cellular senescence and low-grade inflammation favor the acceleration of aging. The liver is an essential metabolic organ because changes related to its function are related to age-related diseases. The objective of this study was to evaluate the effects of maraviroc (MVC) and/or rapamycin (RAPA) on liver tissue in an experimental model of frailty syndrome in mice, since MVC and RAPA are two molecules able to decrease CCR5 expression, which is overexpressed in patients with frailty. Methods: Eighty male homozygous IL10KO mice were randomly assigned to one of 4 groups (n = 20): i) IL10KO group; ii) MVC group, iii) RAPA group, and iv) MVC-RAPA group. Liver samples were analyzed. Gene expression quantification and western blotting were also performed. The proinflammatory cytokines IL-6 and IL-18 were decreased in MVC and MVC/RAPA groups, IL-12 was decreased in RAPA and MVC/RAPA groups and TNF-α was decreased in all therapeutic groups. P21 was decreased in RAPA and MVC/RAPA groups, Galactosidase beta-1, was also significantly reduced in all therapeutic groups, as were NF-kB1, NF-kB2 and STAT3. In all groups, mTOR and CCL5 were significantly reduced. CCR5 expression was decreased in the MVC and MVC/RAPA groups. Conclusion: MVC and RAPA may protect against some factors involved in liver aging. More studies will be necessary to verify their clinical applications.

## Introduction

Aging is directly related to the natural imbalance of the body. Therefore, it reduces survival and, at the same time, increases the risk of disease and death [1]. Aging causes not only a gradual loss of function or degeneration of the organism but also gain of function changes that allow cells to proliferate inappropriately [2]. In this context, aging is a major risk factor for the development of chronic diseases such as neurodegenerative and cardiovascular diseases, diabetes mellitus, osteoporosis, cancer and frailty. Indeed, the risk of developing frailty increases with age, suggesting an association between both processes [3]. Besides, the mechanisms of action of these diseases are closely related to aging [1].

The immunological changes associated with aging are characterized by a chronic low-grade inflammatory state (inflammaging) [4]. This inflammatory phenotype is associated with increases in inflammatory biomarkers such as C-reactive protein (CRP), interleukin-6 (IL-6), or tumor necrosis factor-alpha (TNF-α), which are associated with increased morbidity and mortality in older patients [3, 4].

The liver is a key organ that leads the energy metabolism of our body, connecting metabolic pathways between different tissues, including muscle and adipose tissue [5]. On the other hand, the imbalance of hepatic metabolism favors the development of diseases related to aging, such as insulin resistance, diabetes mellitus and non-alcoholic fatty liver [6, 7].

The development of aging in the liver is favored by genomic alterations and mitochondrial disorders that increase cellular senescence and the appearance of low-grade inflammation [8]. This metabolic damage in the aging liver can favor the cellular senescence of hepatocytes since they present altered genes involved in the glucose and protein synthesis pathway [9]. Therefore, senescent cells increase the activity of senescence-associated β-galactosidase (SA-β-gal), a biomarker related to increased lysosome levels [10]. Studying the levels of beta-galactosidase activity associated with cellular senescence can allow us to quantify the presence of senescent cells in the liver of ageing mice [10]. Moreover, cellular senescence is triggered by the activation of p16 ink4a and p21 Cip1 signaling [11]. In this context, senescent hepatocytes release proinflammatory cytokines such as IL-6 and TNF-α that are related to inflammation and age [12].

IL-10 homozygous knockout (IL-10tm/tm [IL10KO]) mice are an excellent tool for the study of frailty [13], a syndrome associated with aging [3], because they develop sarcopenia, muscular weakness and weight loss [14]. In this mouse model, the inflammatory signaling pathway has been altered by eliminating IL-10 (knockout of IL-10) [15]. This cytokine has anti-inflammatory activities because it suppresses the activation of macrophages and inhibits the production of inflammatory cytokines by Th1 cells [16]. Therefore, since these mice develop a chronic inflammation, they give rise to a mouse model with a disordered liver [17].

Increasing scientific evidence suggests that aging is a regulated process, and its course can be modified by modulating signal transduction pathways [18] including mammalian target of the rapamycin (mTOR), AMP-activated protein kinase (AMPK) [17] and a member of the signal transducer and activator of transcription 3 (STAT3) of signaling molecules [19].

Maraviroc (MVC), a specific C-C chemokine receptor type 5 (CCR5) antagonist, has shown some beneficial effects on certain factors involved in the development of frailty in mice [20]. CCR5 is a receptor that regulates the trafficking and effector functions of memory and effector T lymphocytes, macrophages, and immature dendritic cells [21]. One of CCR5 ligands is C-C motif chemokine ligand 5 (CCL5), which belongs to the CC family of inflammatory chemokine and plays an important role in the frailty syndrome [22] and inflammation [23]. In addition, rapamycin (RAPA), a specific inhibitor of mTOR pathway [23], not only extends the lifespan of aged mice [24], but can also decrease CCR5 mRNA expression [25], which is overexpressed in patients with frailty [26].

Studies by our group examined murine models of liver damage and observed that animals treated with MVC exhibited a better anti-inflammatory profile than control animals [5, 27]. Therefore, in the present study, our objective was to evaluate the effects of MVC and/or RAPA on a disordered liver by using an experimental mouse model of chronic inflammation and frailty, a pathology related to aging.

## Results

In this study, IL-10 deficient mice were randomly assigned to one of the four groups (control, MVC, RAPA or MVC/RAPA) and received the corresponding treatment previously described [28]. All groups had similar survival rates [28]. The four groups had a similar baseline weight and none of the therapeutic interventions reduced body weight. In fact, there were no body weight changes both during and at the end of the experiment (week 24) [28]. We analyzed different parameters in the liver tissue.

The expression of proinflammatory cytokines is decreased after MVC and RAPA treatments

**Fig 1:**
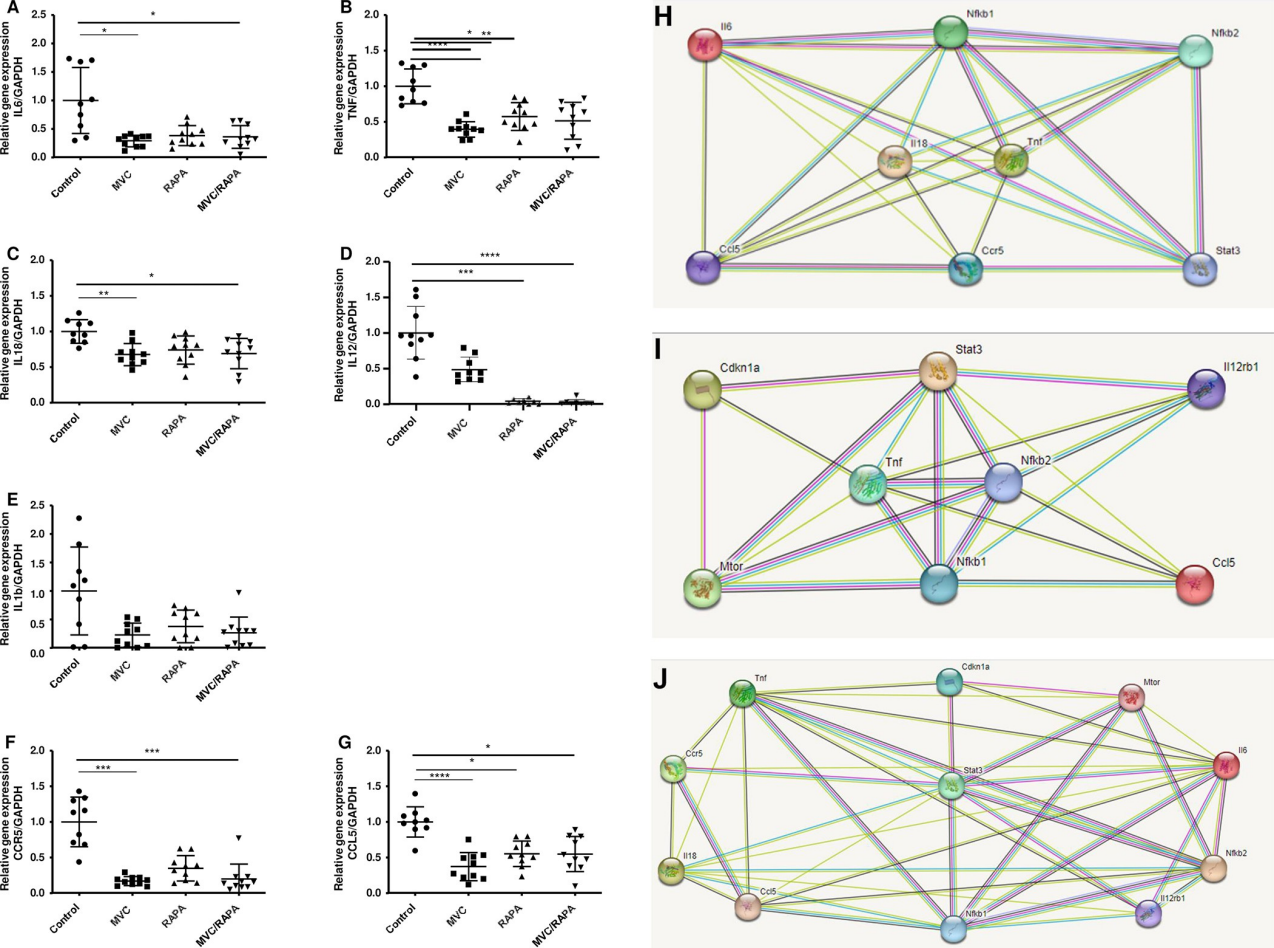
Liver expression of *IL-6*, *TNF-α*, *IL-18*, *IL-12*, *IL-1β*, *CCL5* and *CCR5* at the RNA level and STRING analysis separated by treatment groups.

First, to evaluate the effect of the different treatments in the immune system, we measured the levels of different cytokines. At the mRNA level, compared to that in the control group, the *IL-6* level was significantly lower in the MVC and MVC/RAPA groups (p<0.05 in both). The RAPA group also showed a clear tendency toward lower levels (p = 0.07) (Fig 1A). The expression of *TNF-α* was significantly lower in the MVC (p<0.0001), RAPA (p<0.05) and MVC/RAPA groups (p<0.01) (Fig 1B). In addition, the levels of *IL-18*, another proinflammatory cytokine, were also significantly lower in the MVC (p<0.01) and MVC/RAPA groups (p<0.05) (Fig 1C) and the *IL-12* level was significantly lower in the MVC (p<0.001) and MVC/RAPA (p<0.0001) groups (Fig 1D). *IL-1β*, another proinflammatory cytokine was examined, and the MVC group showed a tendency toward lower levels (p = 0.07) (Fig 1E).

Therefore, the MVC and RAPA treatments and the combination of both (MVC/RAPA) induced a decrease in the mRNA expression of different cytokines. Direct interactions between cytokines *IL-6*, *TNF-α*, *IL-18* and *IL-12* were predicted by STRING analysis (Fig 1H–1J), separated by treatment groups, suggesting an extended phenotype beyond IL10, comprising all immune or inflammatory system.

### The expression of CCR5 and CCL5 biomarkers is decreased after MVC and RAPA treatments

Then, we evaluate the expression of *CCR5*, target of both MVC and RAPA treatments, and its ligand *CCL5*. Compared to that in the control group, *CCL5* mRNA expression was lower in the MVC (p<0.0001), RAPA (p<0.02) and MVC/RAPA groups (p<0.02) (Fig 1F). On the other hand, liver *CCR5* expression was significantly reduced in the MVC (<0.0005) and MVC/RAPA groups (p<0.0001) (Fig 1G). Besides, direct interactions between *CCL5* and *CCR5* were predicted by STRING analysis (Fig 1H–1J).

Therefore, we corroborate that *CCR5* and *CCL5* mRNA expressions were reduced with MVC and RAPA treatments, as expected, since CCR5 is a target of MVC and it is also reduced after RAPA treatment.

### MVC and RAPA treatments also reduced other biomarkers of the inflammatory response

**Fig 2:**
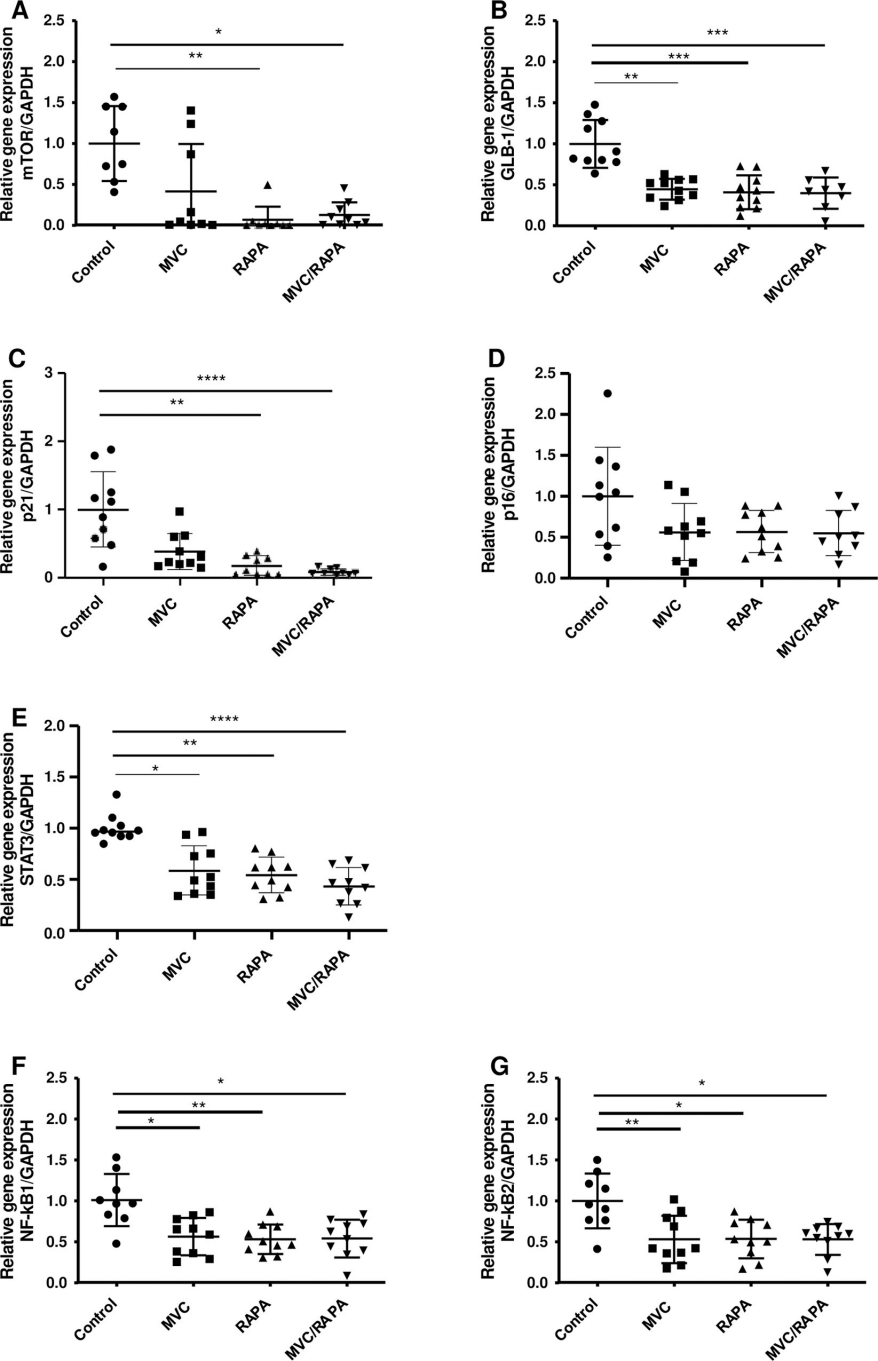
Liver expression of *mTOR*, *GLB-1*, *P21*, *P16*, *STAT3* and *nuclear factor KB 1* and *2* at the mRNA level.

Since RAPA is a specific inhibitor of mTOR pathway [23], we also measured *mTOR* expression levels in the different groups of treatment. mRNA levels of *mTOR* were notably reduced in the RAPA and MVC/RAPA groups (p<0.01 and p<0.05, respectively), and there was a clear trend in the MVC group (p = 0.07) (Fig 2).

To evaluate the presence of senescent cells in the liver tissue, we measured the levels of *Galactosidase beta-1* (*GLB-1*), *p21 Cip1 (P21)* and *p16 ink4a (P16)*. *GLB-1* mRNA levels were also significantly reduced in the MVC (p<0.01), RAPA (p<0.001) and MVC/RAPA groups (p<0.001). All of the treatments seem to reduce the presence of this marker of senescent cells (Fig 2B). In addition, *P21* levels was significantly reduced in the RAPA (p<0.01) and MVC/RAPA groups (p<0.0001) (Fig 2C) and *P16* levels did not showed any significant difference (Fig 2D). In this context, this may indicate that cellular senescence is not promoted after MVC, RAPA or MVC/RAPA treatments.

Since previous reports demonstrated that IL10 repressed inflammation response via activation of *STAT3* [19], we also measured *STAT3* expression levels in the different groups of treatment. The expression of *STAT3* was significantly lower in the MVC (p<0.05), RAPA (p<0.01) and MVC/RAPA groups (p<0.0001) (Fig 2E).

Furthermore, we measured the levels of *NF-kB*, a key transcription factor that regulates the inflammatory response [29]. *NF-kB1* was examined, and the mRNA levels were significantly reduced in the MVC (p<0.05), RAPA (p<0.01) and MVC/RAPA groups (p<0.05). A similar outcome was observed regarding liver expression of *NF-kB2* mRNA in the MVC (p<0.01), RAPA (p<0.05) and MVC/RAPA groups (p<0.05) (Fig 2F and 2G). Therefore, the expression of both *NF-kB1* and *NF-kB2* were reduced after all of the treatments. Direct interactions between others biomarkers: *mTOR*, *GLB-1*, *P21*, *P16*, *STAT3*, *NF-kB1* and *NF-kB2* were predicted by STRING analysis (Fig 1H–1J).

### AKT/mTOR pathway is activated after MVC and RAPA treatments

Next, we measured the expression of some of these markers at the protein level, including their phosphorylated and activates forms.

Mice treated with MVC showed a significant increase in total NF-kB (p <0.05) and the RAPA group showed an increase in p-NF-kB (p<0.01). When NF-kB is activated, it is phosphorylated and translocate to the nucleus to induce the expression of some proinflammatory genes such as IL-1β and TNF-α. Therefore, it seems that NF-kB is activated after MVC and RAPA treatments.

**Fig 3:**
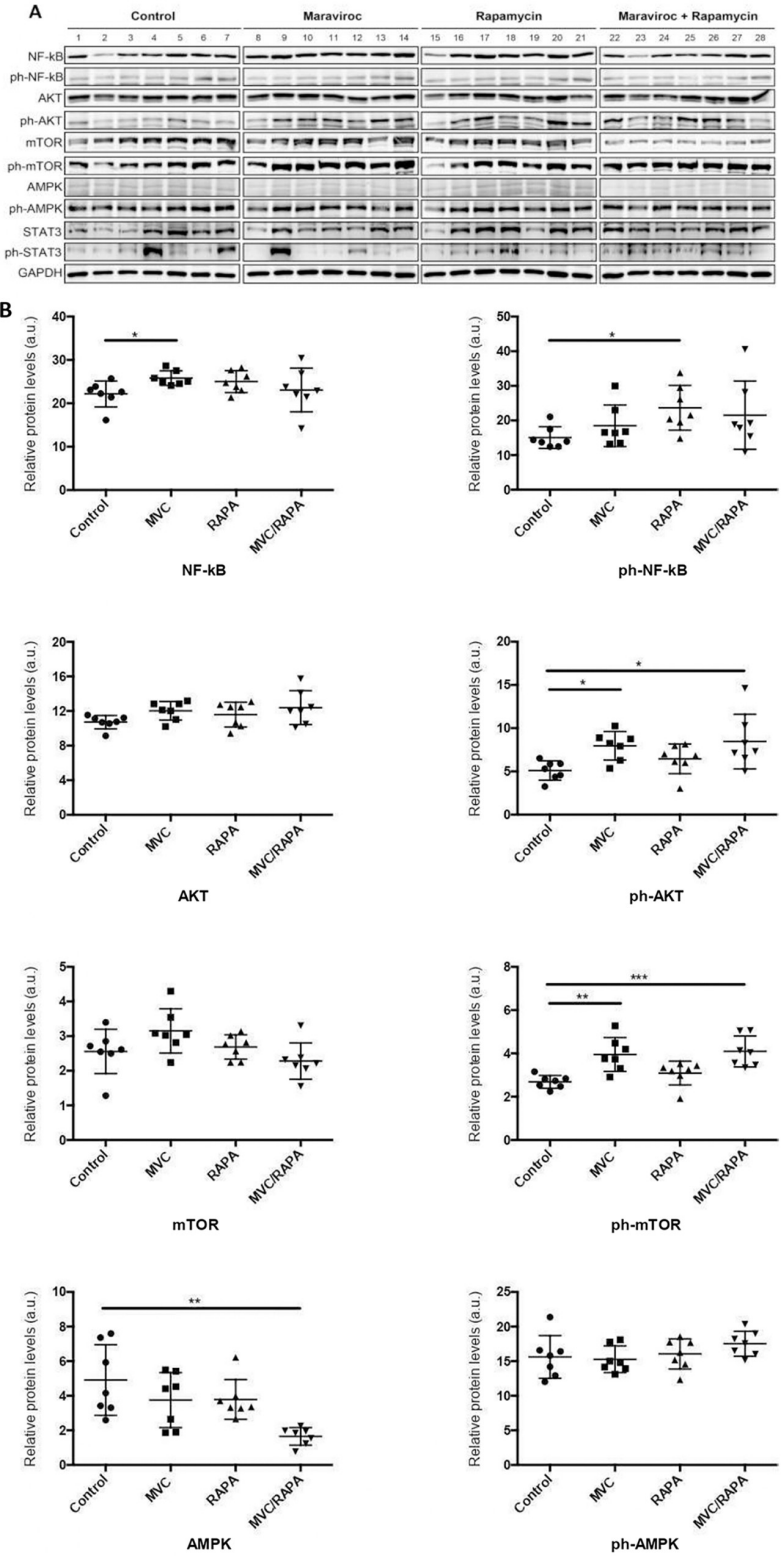
Analyses of the molecular pathways involved in the mechanism of action of MVC and RAPA.

Mice treated with MVC and MVC-RAPA showed significant increase in p-Akt (p <0.05). In the case of mTOR, we observed a decrease when mice were treated with the combination MVC-RAPA, but mice treated with MVC showed an increase in p-mTOR (p<0.01) and the MVC-RAPA group showed a significant increase in p-mTOR (p <0.001). Finally, we observed that mice treated with MVC, RAPA and MVC-RAPA reduced the levels of AMPK, which also inhibits mTOR. On the other hand, STAT3 (total and phosphorylated) was examined but didn´t show significant differences (Fig 3A and 3B and S1 Fig).

Therefore, it seems that AKT/mTOR pathway is activated after MVC and RAPA treatments. Given the specificity of RAPA for mTORC1 [37], and the differential activity to mTORC2, we can suggest that we predominantly detect mTORC2 phosphorylation and activity or some feed-back loop maintaining mTOR phosphorylated even on mTORC1 complex.

## Discussion

Most chronic human diseases increase with age [28]. This makes it important to look for different strategies to slow down aging. It is known that the lifespan of mice can be modified by dietary, genetic, and pharmacological interventions [30]. Exciting findings have shown that rodent aging can be accelerated, stopped, or reversed simply by altering the systemic environment [31]. This growing line of research may offer strategies for treating aging. In this context, RAPA was the first drug to dramatically slow aging in mice [32]. RAPA also increases life expectancy in most studies and protects against many age-related diseases [32]. Other drugs, such as metformin and acarbose, also prolong the lifespan of mice [33, 34]. MVC also has some potential benefits on aging because it modifies certain factors involved in the development of frailty in mice, such as myostatin and certain inflammatory cytokines [28]. In relation to the liver safety of these therapeutic interventions, in a previous study [28] we observed that all of them reduced the transaminases levels.

mTOR has been implicated in many of the processes associated with aging, including cellular senescence, immune responses, stem cell regulation and mitochondrial function [35]. mTOR kinase functions within two multiprotein complexes called mTORC1 and mTORC2 with different subunits and specificities [35]. Inhibition of mTORC1 has prolonged lifespan in all species studied to date and has ameliorated multiple age-related pathologies, including decreased immune function [36].

In addition, some other reports also suggested that the coordination between mTOR and a STAT3 pathway in the modulation of innate immune response [19]. STAT3 was originally identified as a transcription factor that regulates gene expression in response to inflammatory stimulation evoked by ligands whose receptor complexes contain gp130, such as IL-6 [19]. IL-6, as a pro-inflammatory cytokine, it preferentially activates signal transducer and activator of transcription protein 3 (STAT3)-dependent gene expression [37]. Activation of STAT3 by IL-6 plays a crucial role in inflammation-induced disease pathogenesis [38]. On the other hand, it has been demonstrated that, important CCR5-activated signaling proteins such as janus kinase 2 (JAK2) and STAT3 were inhibited by maraviroc [39].

**Fig 4:**
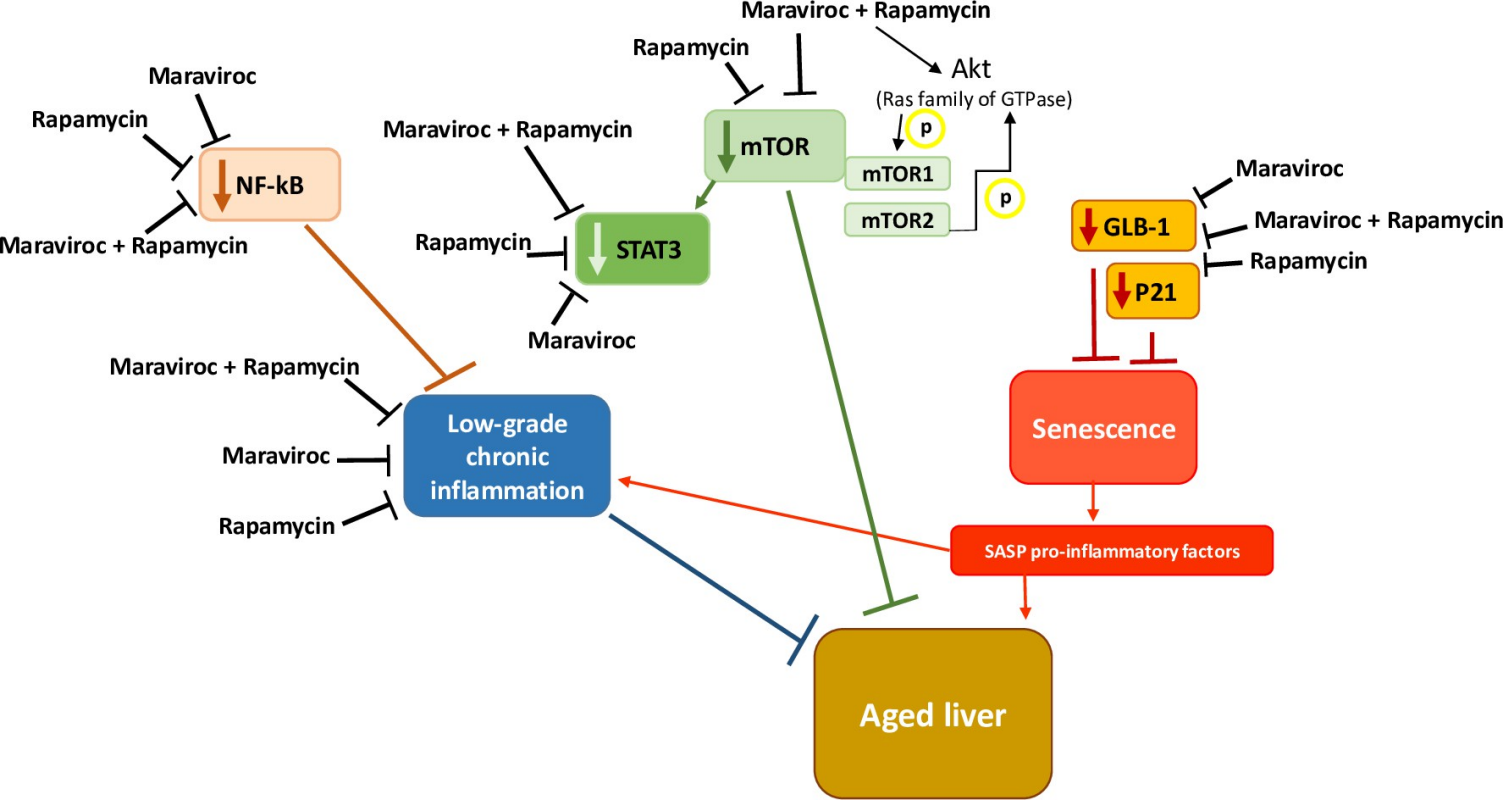
Schematic graph of the postulated mechanism by which MVC and RAPA interfere in an aged mouse liver model.

Inhibiting mTOR with RAPA delays aging and increases lifespan in mice [23]. In our study, MVC, RAPA or MVC-RAPA reduced the mRNA levels of mTOR. However, there is evidence of increased mTOR activity, at least in the liver, in mice in which the aging process has been delayed, such as due to gene mutations [24] (Fig 4). Given the specificity of RAPA for mTORC1 [40], and the differential activity to mTORC2, we can suggest that we predominantly detect mTORC2 phosphorylation and activity, or some feed-back loop maintaining mTOR phosphorylated even on mTORC1 complex. mTORC2 might maintain the phosphorylation of AKT by direct phosphorylation maintaining some feedback loop on mTORC1 phosphorylation, even in the presence of RAPA, which will inhibit further mTORC1, but not mTORC2, activity [40].

In our study, all mice that were treated with MVC also showed higher protein levels of p-mTOR. The phosphorylation of mTOR in mTORC1 by Akt occurs directly via Rheb (Ras family of GTPase) [41]. However, since the mechanism seems the same in both cases, it might be more related to AKT activity than mTOR. There are proteins and transcription factors that can be activated or inhibited after the phosphorylation of the Akt pathway [41–45] without involving mTORC1. The frailty in this model might partly relay in these pathways, like oxidative stress, which has also been related to aging [46, 47]. Nrf2 is a key factor in downstream of PI3K/Akt and is involved in the regulation of oxidative stress and inflammatory response [48]. Therefore, PI3K/Akt pathway may play an important role in the activation of Nrf2. In our study, both MVC and MVC/RAPA activated AKT, and further research with AKT inhibitors [48] or antioxidants, could help to discriminate the effects on the relation between inflammation and frailty syndrome.

In addition to activated Akt, inflammatory and oxidative stress stimulate the NF-kB family of transcription factors [40] (Fig 4). In mouse models, the inhibition of NF-kB has been shown to lead to late onset of age-related symptoms because most of the genes under the transcriptional control of NF-kB are involved in biologic pathways associated with aging, including immune responses, cell senescence, apoptosis and metabolism [40]. All of these factors contribute to age-related tissue degeneration [6]. These changes have been described in liver tissue from mice and rats [8]. Our results showed that at the mRNA level, there was a decrease in NF-kB expression in the liver (1&2) in all therapeutic groups, while at the protein level; there was an increase in total NF-kB expression in the MVC group. This increase was the same as that observed in the muscles of aged IL-10 mice [28]. NF-kB activation is related to many of the known lifespan regulators, including mTOR [49]. Therefore, NF-kB represents a potential antiaging therapeutic target. Consistent with our results, mTOR and NF-kB signaling are coregulated [50] (Fig 1). Another component that contributes to the increase in NF-kB activity that is associated with aging is control of the expression of inflammatory cytokines [51]. Increasingly, inflammation is being linked to aging and chronic diseases [52]. The basal inflammatory response increases with age, leading to low-level chronic inflammation that is likely maladaptive and promotes aging. In this animal model of frailty, we observed a decrease in the mRNA levels of proinflammatory cytokines (IL-6, TNF-α, IL-18 and IL-12) (Fig 1). Therefore, the main protective effect of MVC and RAPA depends on a proinflammatory pathway. This inflammatory phenotype accelerates aging (Fig 4).

Since rapamycin was reported in 2009 to increase the lifespan of mice, there are more than twenty studies published showing that cellular senescence is attenuated by rapamycin [53]. Selvarani R et al. shows a list of different studies in which rapamycin reduces or blocks senescence in a variety of cells from humans, mice, and rats [53]. In addition to suppressing markers of senescence, such as P21 or SA-β-gal-positive cells, rapamycin reduced/prevented the senescent associated secretory phenotype (SASP) phenotype (e.g., the expression and secretion of proinflammatory cytokines by senescent cells such as Il-6 and TNF-α) [51]. This SASP causes DNA damage of sufficient magnitude to induce senescence. Furthermore, SASPs produced by senescent cells could play a role in the age-related increase in chronic inflammation [54, 55]. Therefore, cellular senescence could be an important mechanism underlying aging, because it affects tissue regeneration and drives chronic low-grade inflammation, which exacerbates the aging process [54, 55]. In the same way, in our previous study, we detected at the mRNA muscle level, the expression of IL-6 were significantly lower in the MVC-RAPA group and showed a tendency in the MVC group [28]. Likewise, in muscle samples, IL-18 mRNA levels, another proinflammatory cytokine, were significantly lower in the MVC group [28].

To better understand the mechanism underlying these observations, in this animal model, we did not observe a synergistic, additive or antagonistic effect on the levels of CCR5 or CCL5 mRNA in the liver in the MVC-RAPA group. CCL5 plays an active role in the recruitment of a variety of leukocytes to inflammatory sites [25] (Fig 1). In collaboration with certain cytokines that are released by T cells (e.g., IL-2), CCL5 induces the activation and proliferation of certain natural killer cells to generate chemokine-activated C-C killer cells [56]. CCL5/CCR5 interactions can act as growth factors, inducing the recruitment of additional inflammatory cells and participating in immune evasion [26].

Based on our study, mTOR and NF-kB signaling are coregulated in the liver of IL-10tm/tm [IL10KO] mice, demonstrating that the main protective effect in this animal model of MVC and RAPA could depends on a proinflammatory pathway (Fig 4). This inflammatory phenotype accelerates aging. In summary, our data suggest that the use of MVC and/or RAPA could have protective effects on some factors involved in liver aging. Additional studies will be necessary before justifying a randomized, controlled trial to determine their beneficial effects.

## Material and methods

### Ethics information

All procedures were carried out in accordance with the European Communities Council Directive (86/609/ CEE) on animal experiments and with approval from the ethical committee on animal welfare of our institution (Comité Ético de Experimentación Animal del Centro de Investigación Biomédica de La Rioja, CEEA-CIBIR). In addition, all the study was carried out in compliance with the ARRIVE guidelines (https://arriveguidelines.org). Consent to participate not applicable.

The Materials and Methods section has been published elsewhere [28]. Briefly, a total of 80 male homozygous IL-10-deficient mice (B6.129P2-IL10tm1Cgn/J) were purchased from Jackson Laboratory (Bar Harbor, ME, USA). When the animals were approximately 6 weeks old, they were randomly assigned (n = 20) to one of 4 groups and fed for 24 weeks: i) the IL-10KO group (IL-10KO) received a standard rodent diet and tap water; ii) the preventive MVC group received the same diet as the IL-10KO group and were administered MVC (Pfizer, New York, N York) in their drinking water (300 mg/L) [6, 26, 28]; iii) the preventive RAPA group received the same diet as the IL-10KO group and were administered RAPA in their drinking water (1.5 mg/kg/day) [28]; and iv) the preventive MVC plus RAPA group (MVC-RAPA) received the same diet as the IL-10KO group and were administered MVC plus RAPA in their drinking water at the same concentration as in the MVC and RAPA groups.

The mice were observed daily, and all observations were recorded. All animals were sacrificed on week 24 by CO_2_ exposure. Blood samples were collected under anesthesia after a 4-hour fasting period. Internal organs were examined macroscopically and weighed.

### Gene expression quantification

Total RNA was extracted and purified from liver samples using an RNA RNeasy Mini Kit (Qiagen, Valencia, CA) and treated with DNase I (Qiagen) according to the manufacturer’s instructions [28]. cDNA was synthesized by reverse transcription of 1 μg of total RNA using the SuperScript III First-Strand Synthesis kit (Invitrogen, Carlsbad) in a total volume of 20 μl according to the manufacturer’s instructions, followed by amplification using SYBR Green (Takara Bio Inc, Shiga, Japan) The PCR primer sequences are listed in S1 Table. The amplification and detection of specific products were performed using an ABI PRISM 7300 system (Applied Biosystems, Foster City, CA, USA). All reactions were run in duplicate for each sample. The expression of respective genes was normalized to glyceraldehyde 3-phosphate dehydrogenase (GAPDH) as an internal control [28]. All gene interactions were investigated using the STRING database [57].

### Western blot analysis

Liver samples were lysed using a homogenizer in RIPA lysis buffer (Sigma Aldrich, St. Louis, MO). The cell lysate was centrifuged at 10,000 g for 10 minutes at 4°C [28]. The concentration of total protein in each sample was determined by the Bradford method. 5´adenosine monophosphate-activated protein kinase (AMPK) [58], phosphorylated AMPK (pAMPK) [59], protein kinase-B (Akt) [60], phosphorylated Akt (pAkt) [61], nuclear factor-kB (NF-kB) [62], phosphorylated NF-kB (pNF-kB) [63], mammalian target of (mTOR) [64], phosphorylated mTOR (pmTOR) [65], signal transducer and activator of transcription 3 (STAT3) [66] and phosphorylated STAT3 [66] were evaluated by Western blotting (WB). GAPDH was used as an internal control [67] S2 Table.

Proteins were analyzed by colorimetry using a secondary antibody bound to peroxidase (anti-rabbit or mouse IgG, Cell Signaling, Danvers, MA) and incubated with the corresponding substrate [28]. After high-resolution scanning, the concentration of each protein was evaluated by densitometry using Image J software. The density values for each of the test samples were normalized to the values of GAPDH, which was used as a loading control [18].

### Statistical analysis

The data are presented in the figures as the mean ± SE (standard error of the mean). For all data, the Kruskal–Wallis test was used followed by the Mann–Whitney U test. Correlations between variables were determined using the Spearman rank-sum test. We used two-way ANOVA for independent sample comparison. All data were analyzed with GraphPad Prism 6 software and were considered statistically significant when p<0.05.

## Supporting information

S1 Table List of primers employed by SybrGreen for real-time PCR. (PDF)

S2 Table Kinases employed in this study. (PDF)

S1 Fig Original blots of the western blot analysis of Fig 3. (PDF)
