# Supplementary material for: Role of maraviroc and/or rapamycin in the liver of IL10 KO mice with frailty syndrome
Source: PLoS One. 2024 Jan 10;19(1):e0286201. doi: 10.1371/journal.pone.0286201 (PMC10781157; doi:10.1371/journal.pone.0286201)
Supplement: S1 Table — (PDF) [file pone.0286201.s001.pdf]

S1 Table. List of primers employed by SybrGreen for real-time PCR.

Name	Gene symbol	Primers Sequence
**Glyceraldehide 3-phoshate dehydrogenase**	**GAPDH**	Sense: 5´-CATGTTCCAGTATGACTCCACTC-3´<br>Antisense: 5´-GGCCTCACCCCATTTGATGT-3´
**Interleukin-6**	**IL-6**	Sense: 5´-ATGGATGCTACCAAACTGGAT-3´<br>Antisense: 5´-TGAAGGACTCTGGCTTTGTCT-3´
**Interleukin-18**	**IL-18**	Sense: 5´- GACTCTTGCGTCAACTTCAAGG -3´<br>Antisense: 5´- CAGGCTGTCTTTTGTCAACGA -3´
**Tumor necrosis factor- α**	**TNF-α**	Sense 5´- ACGGCATGGA TCTCAAAGAC-3´<br>Antisense 5´- AGATAGCAAATCGGCT GACG-3´
**Interleukin-12**	**IL-12**	Sense 5´- TTATGTTGTAGAGGTGGACTGG-3´<br>Antisense 5´- TTTCTTTGCACCAGCCATGAGC-3´
**Interleukin-1 beta**	**IL-1β**	Sense: 5′-CTGAACTCAACTGTGAAATGCCA-3′<br>Antisense: 5′-AAAGGTTTGGAAGCAGCCCT-3′
**Chemokine receptor type 5**	**CCR5**	Sense: 5´-CGAAAACACATGGTCAAACG-3´<br>Antisense: 5´-TTCCTACTCCCAAGCTGCAT-3´
**Chemokine ligand 5 (RANTES)**	**CCL5**	Sense: 5´-ATATGGCTCGGACACCACTC-3´<br>Antisense: 5´-GTGACAAACACGACTGCAAGA -3´
**Mammalian target of Rapamycin**	**mTOR**	Sense: 5´- CTGGGACTCAAATCTCTCCAGTTC-3´<br>Antisense: 5´- GAACAATAGGGTGAATGATCCGGG-3´
**Galactosidase Beta-1**	**GLB-1**	Sense: 5´-GGATGGACAGCCATTCCGAT-3´<br>Antisense: 5´-CAGGGCACGTACATCTGGATA-3´
**p16 ink4a**	**P16**	Sense 5´- CCCAACGCCCCGAACT-3´<br>Antisense 5´- GCAGAAGAGCTGCTACGTGA -3´
**p21 Cip1**	**P21**	Sense 5´- GTCAGGCTGGTCTGCCTCCG -3´<br>Antisense 5´- CGGTCCCGTGGACAGTGAGCAG -3´
**Signal transducer and activator of transcription 3**	**STAT3**	Sense 5´- CCCCGTACCTGAAGACCAAGT -3´<br>Antisense 5´- CCGTTATTTCCAAACTGCATCA -3´
**Nuclear factor NF-kappa-B p105 subunit**	**Nf-kB1**	Sense: 5´-GAAATTCCTGATCCAGACAAAAAC-3´<br>Antisense: 5´-ATCACTTCAATGGCCTCTGTGTAG-3´
**Nuclear factor NF-kappa-B p100 subunit**	**Nf-kB2**	Sense: 5´-CTGGTGGACACATACAGGAAGAC-3´<br>Antisense: 5´-ATAGGCACTGTCTTCTTTCACCTC-3´
