# Supplementary material for: Role of maraviroc and/or rapamycin in the liver of IL10 KO mice with frailty syndrome
Source: PLoS One. 2024 Jan 10;19(1):e0286201. doi: 10.1371/journal.pone.0286201 (PMC10781157; doi:10.1371/journal.pone.0286201)
Supplement: S2 Table — (PDF) [file pone.0286201.s002.pdf]

S2 Table. Kinases employed in this study.

Antibody used	Commercial Producer	Used dilution	Molecular weight of the protein	Secondary antibody and used dilution
**Anti AMPK** **(Mouse monoclonal)**	Abcam, Cambridge, MA, USA (Cat. No. 80039)	1:1000	62 kDa	Mouse 1:5000
**Anti AMPK (phospho Thr 172)** **(Rabbit monoclonal)**	Cell Signaling, Danvers, MA (Cat. No. 109458)	1:1000	60 kDa	Rabbit 1:5000
**Anti Akt** **(Rabbit polyclonal)**	Cell Signaling, Danvers, MA (Cat. No. 9272)	1:1000	60 kDa	Rabbit 1:5000
**Anti Akt (phospho S473)** **(Rabbit monoclonal)**	Abcam, Cambridge, MA, USA (Cat No. 81283)	1:5000	56 kDa	Rabbit 1:5000
**Anti NF-kB** **(Rabbit polyclonal)**	Abcam, Cambridge, MA, USA (Cat No. 16502)	1:2000	64 kDa	Rabbit 1:5000
**Anti NF-kB (phospho S529)** **(Rabbit monoclonal)**	Abcam, Cambridge, MA, USA (Cat No. 109458)	1:1000	60 kDa	Rabbit 1:5000
**mTOR** **(Rabbit monoclonal)**	Cell Signaling, Danvers, MA (Cat. No. 2983T)	1:1000	289 kDa	Rabbit 1:5000

**Anti mTOR (phospho S2448) (Rabbit monoclonal)**	Abcam, Cambridge, MA, USA (Cat No. 109268)	1:1000	289 kDa	Rabbit 1:5000
**Anti STAT3 (Rabbit monoclonal)**	Abcam, Cambridge, MA, USA (Cat No. 68153)	1:1000	75-88 kDa	Rabbit 1:1000
**Anti STAT3 (phospho Y705) (Rabbit monoclonal)**	Abcam, Cambridge, MA, USA (Cat No. 76315)	1:1000	88 kDa	Rabbit 1:2000
**Anti GAPDH (Mouse monoclonal)**	Thermo Fisher Scientific, Waltham, MA (Cat. No.AM4300)	1.2000	37 kDa	Mouse 1:5000
