# Supplementary material for: Role of maraviroc and/or rapamycin in the liver of IL10 KO mice with frailty syndrome
Source: PLoS One. 2024 Jan 10;19(1):e0286201. doi: 10.1371/journal.pone.0286201 (PMC10781157; doi:10.1371/journal.pone.0286201)

S1 Fig: Original blots of the western blot analysis of Figure 3.

Western blot analysis of the protein levels of NF-KB (total and phosphorylated), AKT (total and phosphorylated), mTOR (total and phosphorylated), AMPK (total and phosphorylated), STAT3 (total and phosphorylated) and GAPDH as endogenous control, in mice treated with Maraviroc (MVC group), Rapamycin (RAPA group) or combination of both (MVC-RAPA group). Blots are grouped in control (G1), Maraviroc (G2), Rapamycin (G3), Maraviroc and Rapamycin (G4). Molecular size control and positive controlare included. Blots for AMPK also include fat samples. All samples derive from the same experiment and gels/blots were processed in parallel.

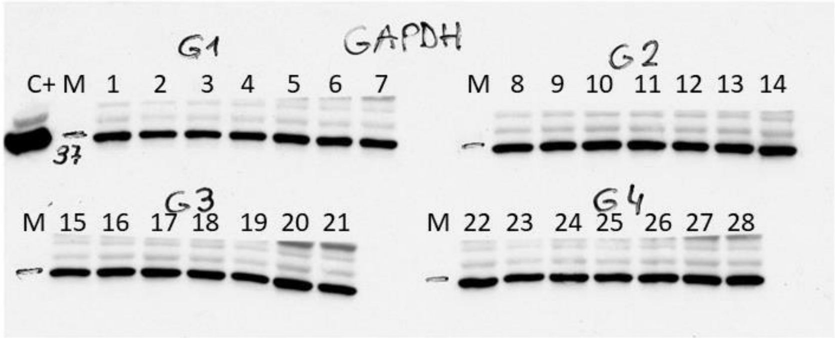

G1 = Control
G2 = Maraviroc
G3 = Rapamycin
G4= Maraviroc + Rapamycin
M=molecular size control
C+= positive control

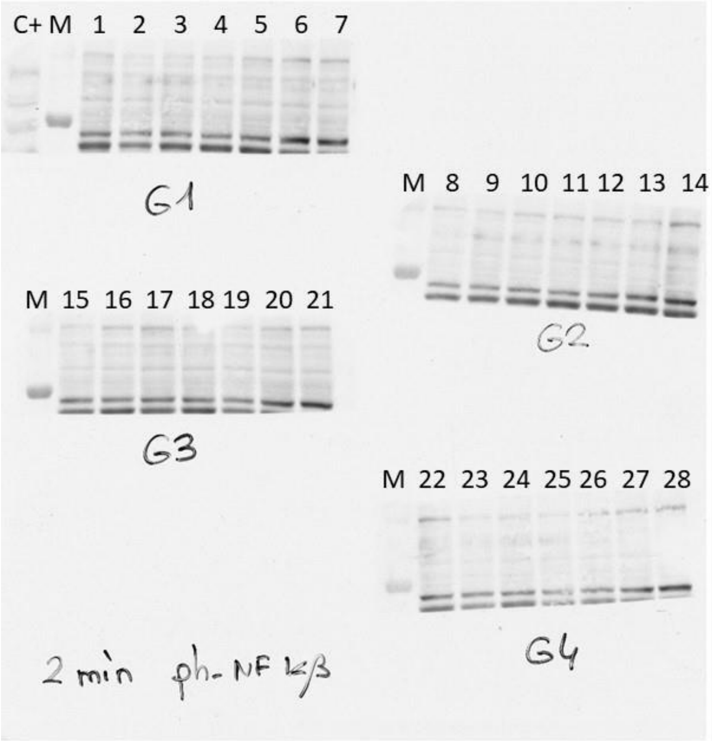

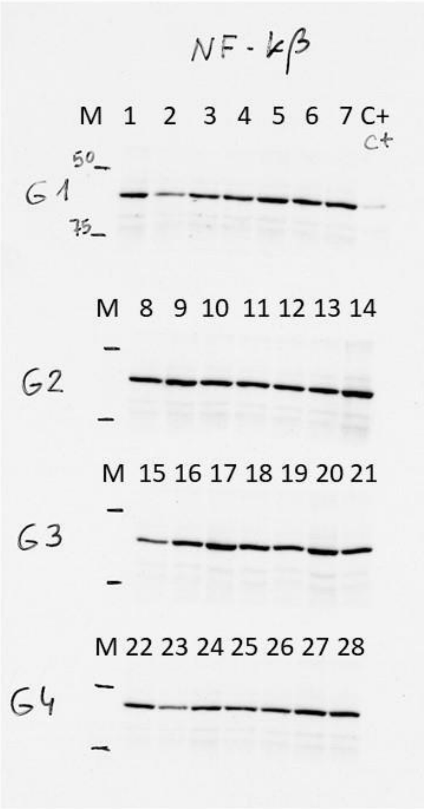

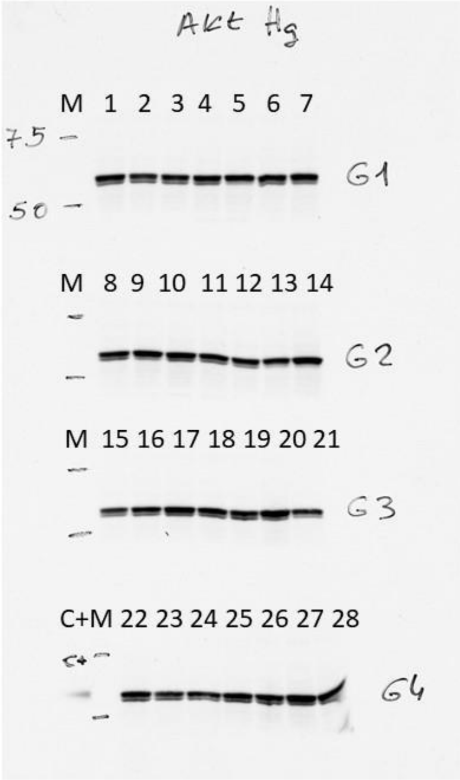
Akt Hg
M 1 2 3 4 5 6 7
75
50
G1
M 8 9 10 11 12 13 14
G2
M 15 16 17 18 19 20 21
G3
C+M 22 23 24 25 26 27 28
C+
G4

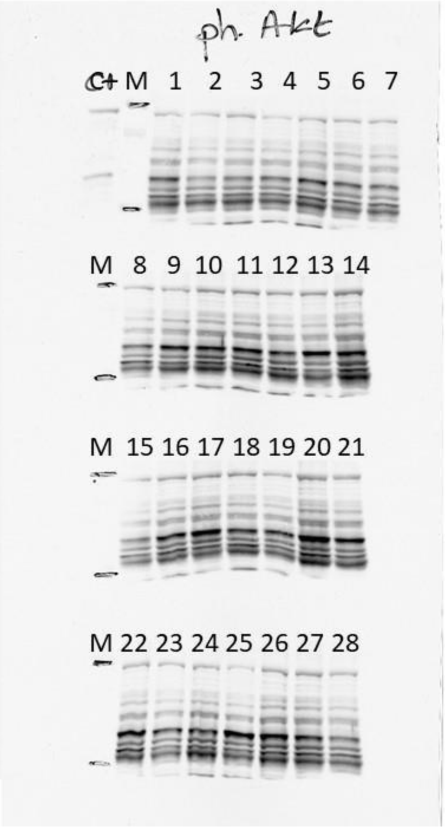
ph. Akt
C+M 1 2 3 4 5 6 7
M 8 9 10 11 12 13 14
M 15 16 17 18 19 20 21
M 22 23 24 25 26 27 28

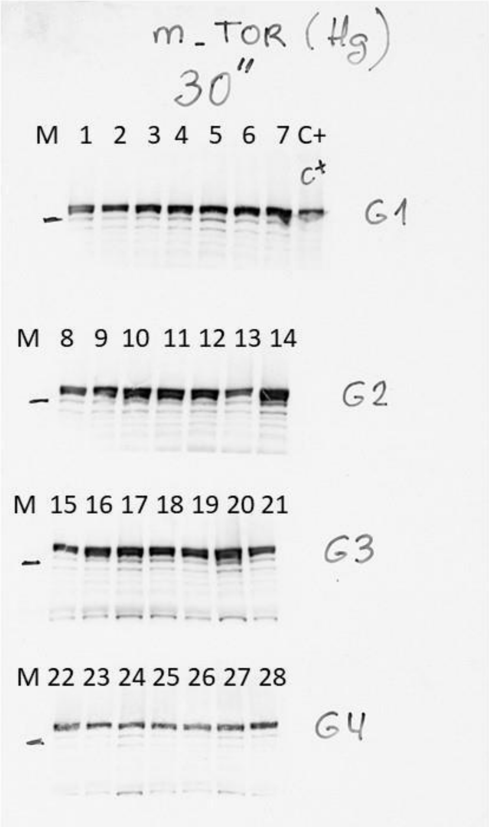
m_TOR (Hg)
30"
M 1 2 3 4 5 6 7 C+
C+
G1
M 8 9 10 11 12 13 14
G2
M 15 16 17 18 19 20 21
G3
M 22 23 24 25 26 27 28
G4

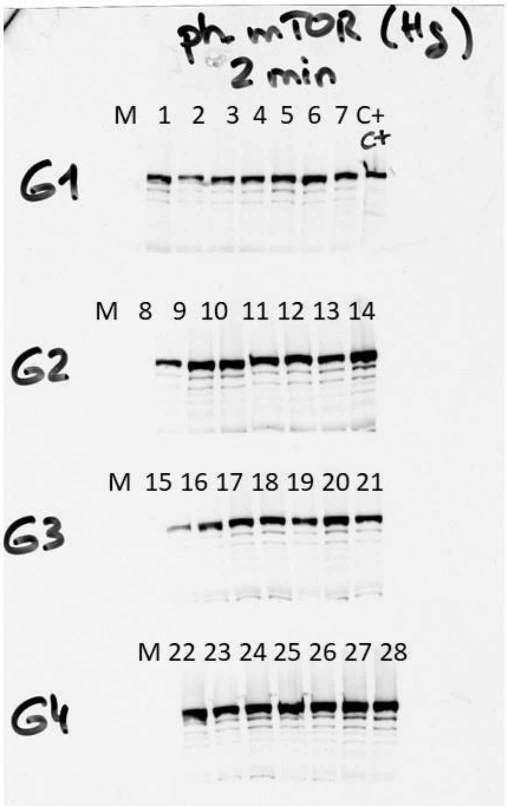
ph. mTOR (Hg)
2 min
M 1 2 3 4 5 6 7 C+
C+
G1
M 8 9 10 11 12 13 14
G2
M 15 16 17 18 19 20 21
G3
M 22 23 24 25 26 27 28
G4

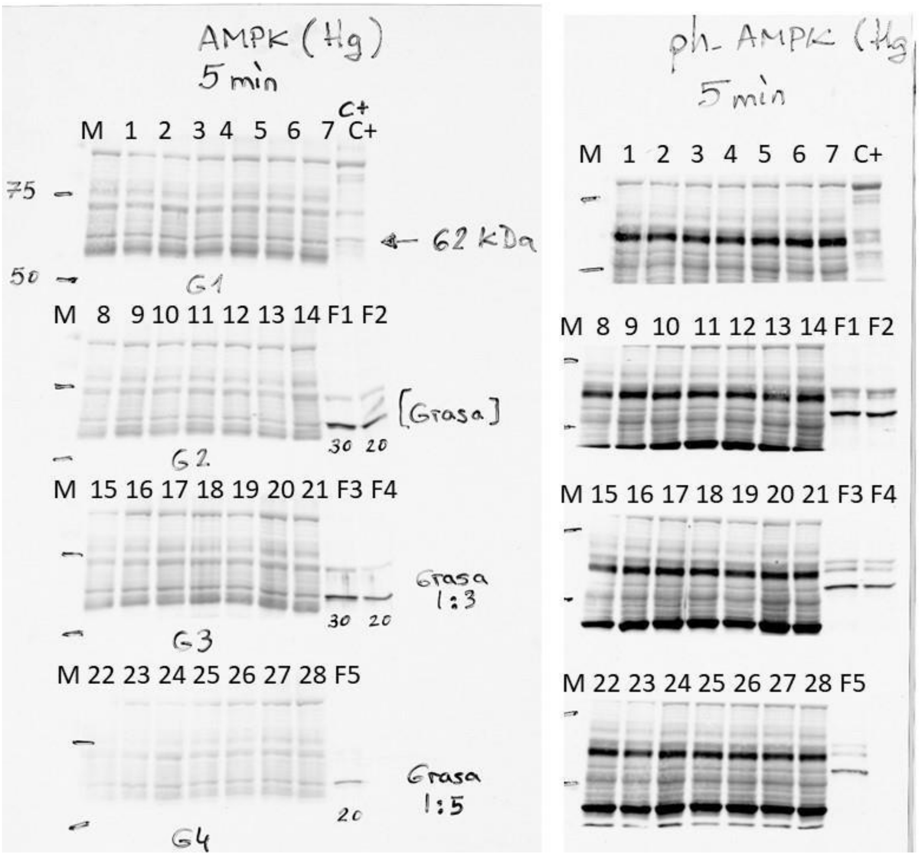

M=molecular size control
C+= positive control
F=fat samples

Original blots for GAPDH (expected band 37KDa)

G1 = Control

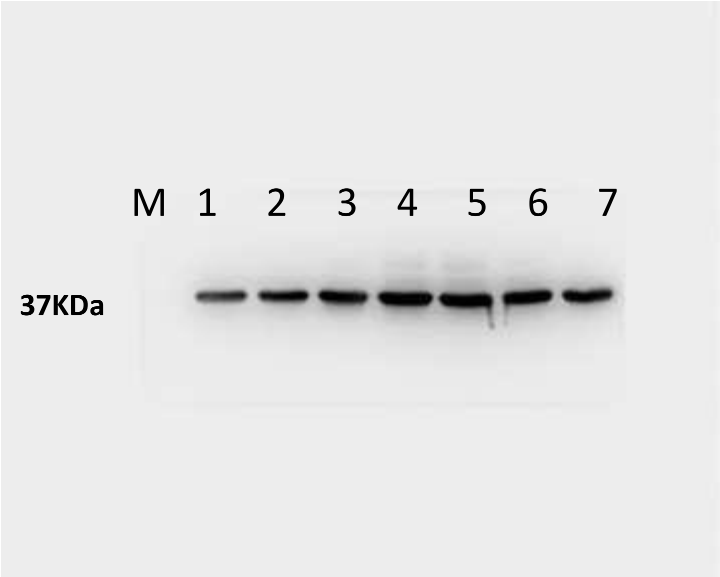

G2 = Maraviroc

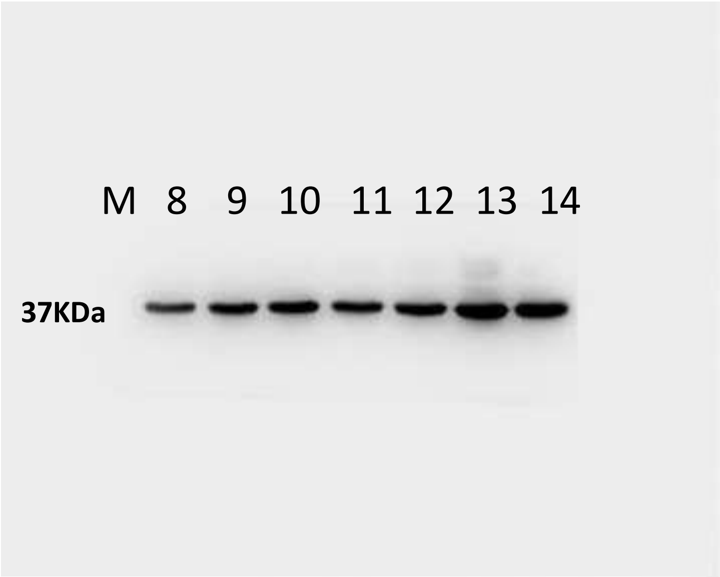

G3 = Rapamycin

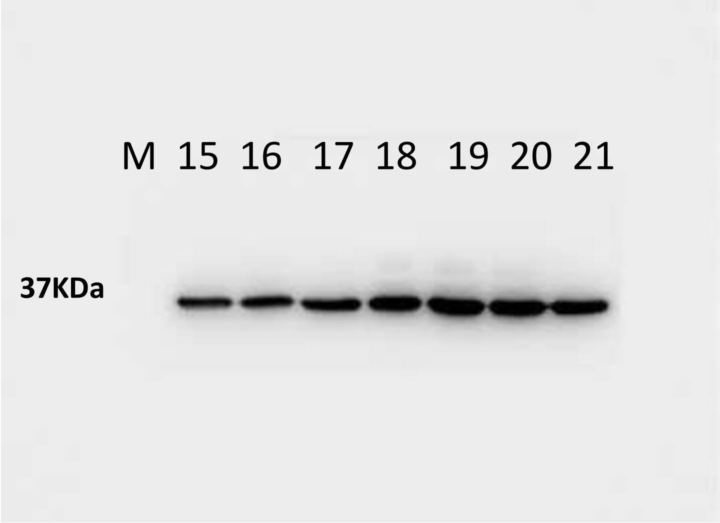

G4 = Maraviroc+Rapamycin

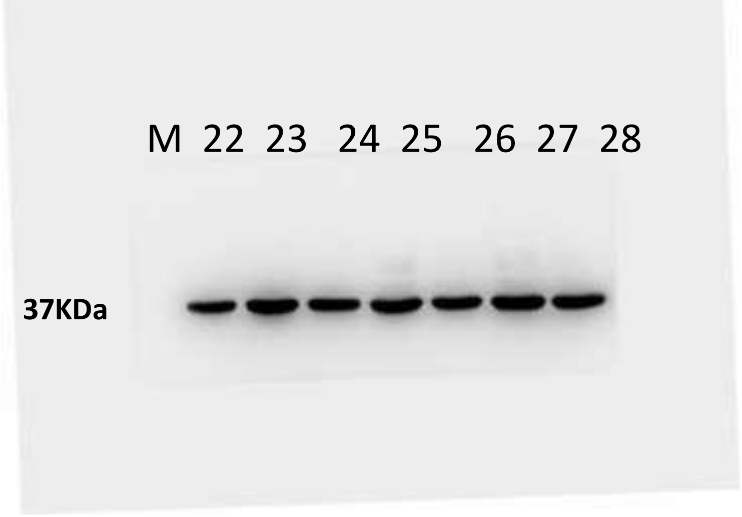

Original blots for STAT3 (expected band 88KDa)

G1 = Control

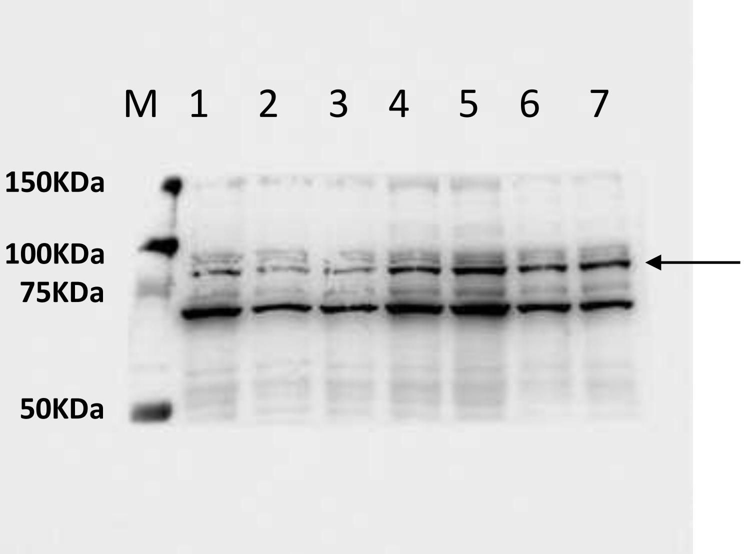

G2 = Maraviroc

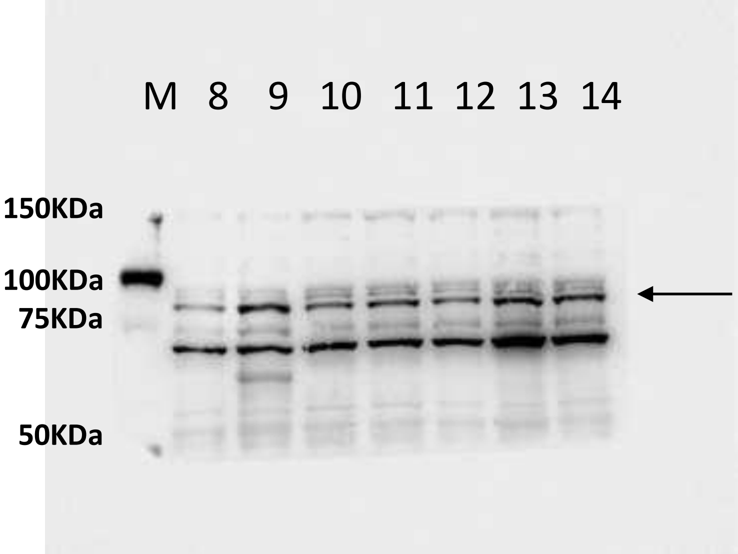

G3 = Rapamycin

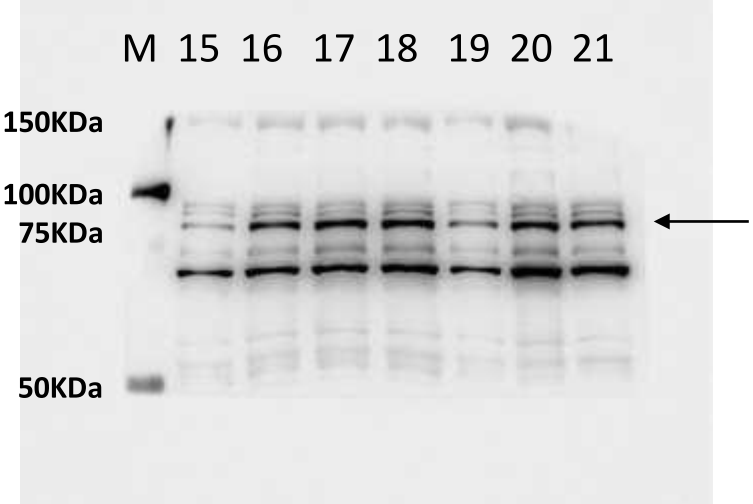

G4 = Maraviroc+Rapamycin

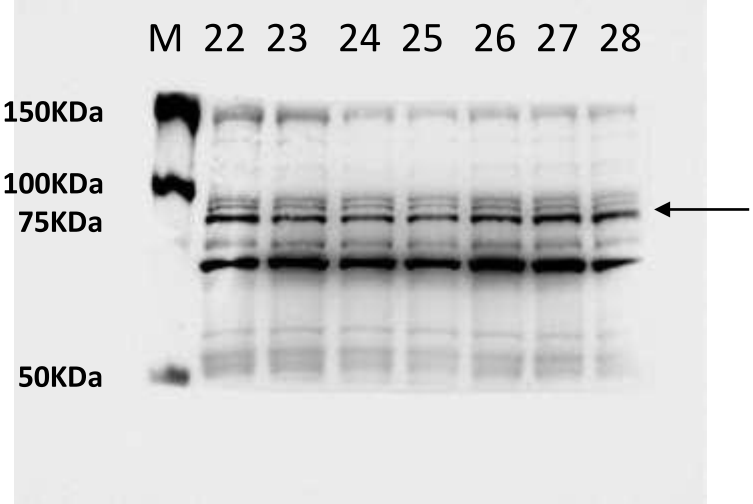

Original blots for ph-STAT3 (expected band 88KDa)

G1 = Control

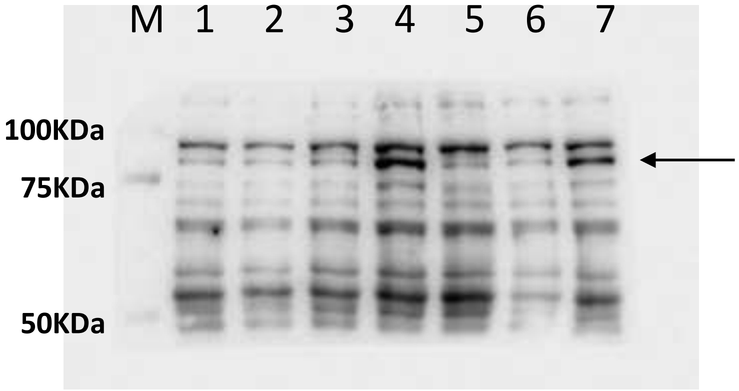

G2 = Maraviroc

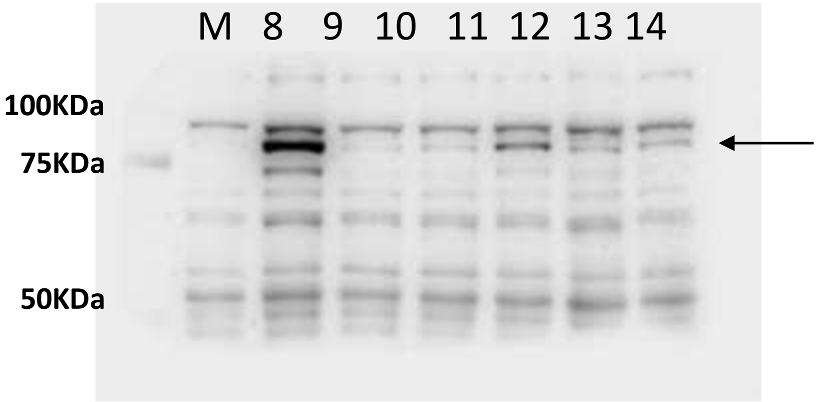

G3 = Rapamycin

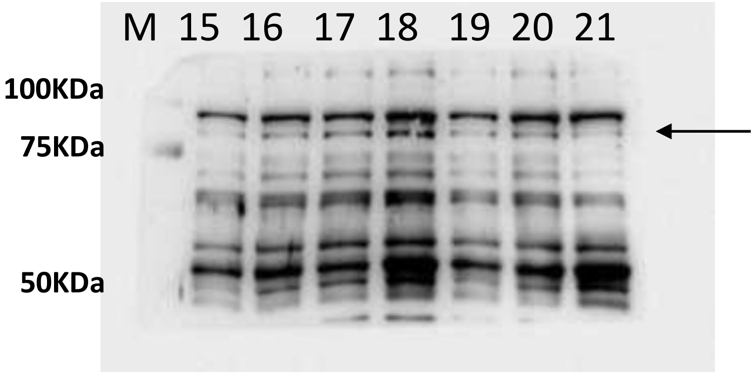

G4 = Maraviroc+Rapamycin

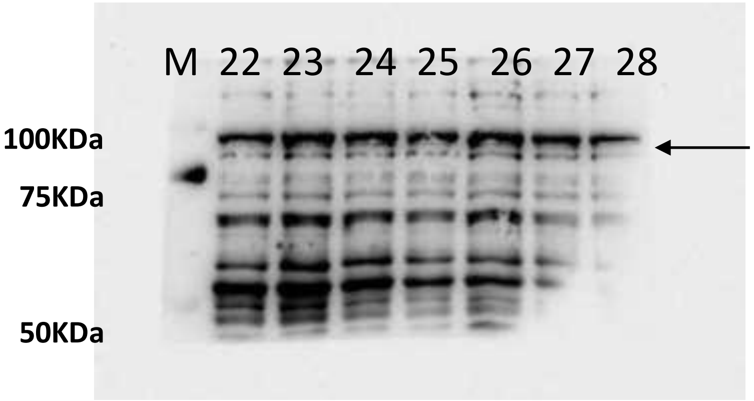

Supplement: S1 Fig — (PDF) [file pone.0286201.s003.pdf]
